# Prostaglandin E2/EP Signaling in the Tumor Microenvironment of Colorectal Cancer

**DOI:** 10.3390/ijms20246254

**Published:** 2019-12-11

**Authors:** Rei Mizuno, Kenji Kawada, Yoshiharu Sakai

**Affiliations:** Department of Surgery, Graduate School of Medicine, Kyoto University, Kyoto 606-8507, Japan; reimzn@kuhp.kyoto-u.ac.jp (R.M.); ysakai@kuhp.kyoto-u.ac.jp (Y.S.)

**Keywords:** colorectal cancer, PGE2/EP signaling, tumor microenvironment

## Abstract

The number of colorectal cancer (CRC) patients is increasing worldwide. Accumulating evidence has shown that the tumor microenvironment (TME), including macrophages, neutrophils, and fibroblasts, plays an important role in the development and progression of CRC. Although targeting the TME could be a promising therapeutic approach, the mechanisms by which inflammatory cells promote CRC tumorigenesis are not well understood. When inflammation occurs in tissues, prostaglandin E2 (PGE2) is generated from arachidonic acid by the enzyme cyclooxygenase-2 (COX-2). PGE2 regulates multiple functions in various immune cells by binding to the downstream receptors EP1, EP2, EP3, and EP4, and plays an important role in the development of CRC. The current therapies targeting PGE2 using non-steroidal anti-inflammatory drugs (NSAIDs) or COX-2 inhibitors have failed due to the global prostanoid suppression resulting in the severe adverse effects despite the fact they could prevent tumorigenesis. Therefore, therapies targeting the specific downstream molecules of PGE2 signaling could be a promising approach. This review highlights the role of each EP receptor in the TME of CRC tumorigenesis and their therapeutic potential.

## 1. Introduction

Colorectal cancer (CRC) is one of the most common causes of cancer-related deaths worldwide [1,2] and the number of CRC patients is increasing progressively [3,4]. A number of studies have revealed the molecular mechanisms of CRC tumorigenesis, including adenoma-carcinoma sequence, de novo carcinogenesis, and inflammation-related carcinogenesis [5]. Inflammatory bowel disease (IBD) is defined as a chronic intestinal inflammation in susceptible individuals influenced by environmental risk factors. Studies have revealed that IBD patients are at high risk of gaining CRC [6,7]. IBD patients are 2–6 times more likely to develop CRC compared with the general population and the number of IBD patients is expected to increase over time [8,9]. Therefore, it is important to understand the mechanisms of inflammation-related carcinogenesis to overcome CRC.

The intestine covers a large part of the body surface contacting with the external environment, and has a function as a barrier against the various harmful substances and pathogens. When the balance between intestinal barrier function and external pathogens collapses, inflammation occurs in the intestine. When inflammation occurs in the intestine, various inflammatory cells such as neutrophils, macrophages and fibroblasts, are recruited by a variety of factors produced by damaged cells. In turn, the recruited inflammatory cells can produce various cytokines and chemokines in response to the inflammation.

**Figure 1:**
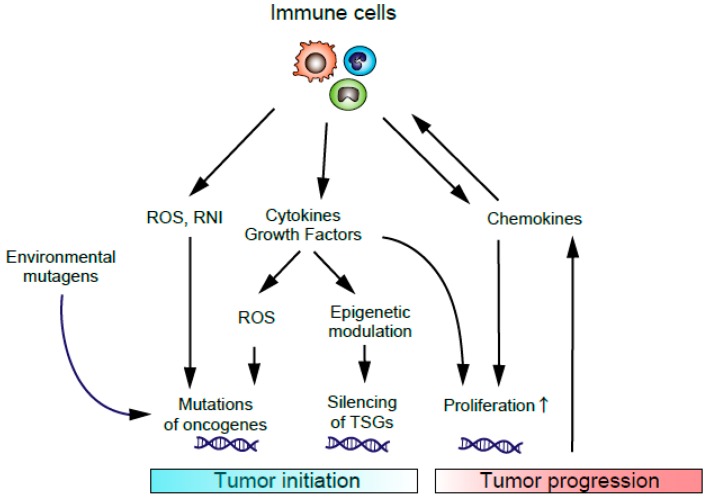
Inflammation-related carcinogenesis. Environmental mutagens, ROS, and RNI produced by recruited immune cells can cause DNA damage, resulting in the initiation of inflammation-related carcinogenesis. Cytokines or growth factors produced by immune cells can induce epigenetic changes in tumor suppressor genes (TSGs) and promote tumor initiation. Cytokines or chemokines from immune cells also promote tumor growth and progression.

When the inflammation occurs in the intestine, fibroblasts and inflammatory cells infiltrate into the inflamed tissues and function within the tumor microenvironment (TME). These cells interact with CRC cells through various cytokines and chemokines to promote tumor growth and progression [10,11,12,13]. In the early stage of tumorigenesis, inflammation-related carcinogenesis, tumor initiation is induced by the DNA damage caused by reactive oxygen species (ROS) and reactive nitrogen intermediates (RNI) produced by the infiltrated immune cells and other mutagens. Cytokines released from infiltrated inflammatory cells can also increase the levels of ROS and RNI in pre-malignant epithelial cells, which changes the epigenetic modification of tumor suppressor genes (TSGs) resulting in the promotion of tumor initiation. In the later stage of tumorigenesis, cytokines and chemokines can support tumor growth by promoting angiogenesis and suppressing anti-tumor immune response (Figure 1). Other factors in the intestine, such as intestinal microbiota and dietary compounds, can influence colon cancer development. Persistent inflammation facilitates tumor promotion by activating proliferation and antiapoptotic properties of tumor cells [14]. 

Prostaglandins are inflammatory mediators which are generated from arachidonic acids by the enzyme cyclooxygenase-2 (COX-2) and also play pleiotropic roles at inflammatory sites [15,16]. For example, prostaglandin E2 (PGE2), a major cyclooxygenase product in several physiological settings, regulates multiple functions of various immune cells [17,18]. Enzymatic degradation of PGE2 involves the NAD+-dependent 15-hydroxyprostaglandin dehydrogenase (15-PGDH). Otani et al., reported that the levels of 15PGDH are reduced in IBD resulting in the increased expression of PGE2 which could worsen the chronic inflammation [19]. PGE2 is also known to promote CRC growth and progression [20]. Recent studies have demonstrated that decreased 15-PGDH has a profound relationship with carcinogenesis and cancer progression in CRCs, breast cancer, prostate cancer, lung cancer, gastric cancer and other cancers [21,22,23]. 

PGE2 transduces its signals by binding to the four PGE2-sensitive (EP) receptors, EP1 to EP4 [24]. The final output of PGE2 signaling depends on the expression of each EP receptor and on the strength of each EP signal. It is known that PGE2 affects not only CRC cells but also inflammatory cells and fibroblasts, because these cells express EP receptors. 

Previous studies have reported that the inhibition of PGE2 by non-steroidal anti-inflammatory drugs (NSAIDs) or COX-2 selective inhibitors could suppress CRC development and progression [25,26]. Although clinical trials investigated the effectiveness of NSAIDs or COX-2 inhibitors for the prevention of CRC, the studies failed due to severe cardiovascular toxicity [27,28]. Therefore, recent studies have been focusing on the more specific downstream signaling of PGE2, especially EP signaling. 

Although accumulating evidence has shown the role of PGE2/EP signaling in CRC tumorigenesis, PGE2/EP signaling in the TME is not fully understood despite the abundant expression of EP receptors in fibroblasts and inflammatory cells. Therefore, in this review, we highlight the role of PGE2/EP signaling in the TME of CRC tumorigenesis.

## 2. PGE2/EP Signaling in CRC Cells

Prostaglandins at the inflammatory sites play pleiotropic roles in inflammation [15,16]. In particular, PGE2, a major cyclooxygenase product in various several physiological settings, regulates multiple functions of immune cells [17,18]. PGE2 signals through four pharmacologically distinct membrane receptors, EP1, EP2, EP3, and EP4, which belong to the G protein-coupled receptor (GPCR) family. Each receptor is coupled to different intracellular signaling pathways, and has distinct biochemical properties and tissue localization [29]. The main signal transduction of PGE2 consists of a rise in intracellular free calcium ion concentration via EP1, a decrease in intracellular cyclic adenosine monophosphate (cAMP) concentration and extracellular signal-regulated kinase (ERK) activation via the inhibitory subunit Gi in EP3, and a rise in intracellular cAMP concentration and subsequent protein kinase A (PKA) activation via the stimulatory subunit Gs in EP2 and EP4 [24]. 

PGE2 signaling affects the biology of intestinal epithelial cells in the physiological condition. Its cellular targets and the resulting physiological changes are predominantly determined by the distribution of EP receptors. Takafuji et al., assessed the distribution of each EP receptor in the normal and inflamed human colon, and found that EP2 and EP3 were expressed on epithelial cells at the apex of crypts, while EP4 was expressed on surface and lateral crypt epithelial cells in normal mucosa. On the other hand, in inflamed intestine, lateral epithelial cells expressed EP2 and EP3 [30]. Other studies using rodents showed that EP1 receptors were expressed in goblet cells in small intestine and in other epithelial cell types in colon [31]. Houchen et al., reported that the expression of EP2 receptors depended on the differentiation state of the epithelial cells. In small intestine, undifferentiated crypt epithelial cells predominantly expressed EP2 receptors on their nuclear membranes, whereas highly differentiated epithelial cells at the apex of the villi expressed these receptors on their plasma membrane [32]. 

A number of studies have revealed that the PGE2/EP signaling in the tumor cells contributes to the CRC tumorigenesis. Watanabe et al. reported that EP1 deficiency decreased the formation of aberrant crypt foci (ACF) which are putative pre-neoplastic lesions in the azoxymethane (AOM)-treated colonic tumorigenesis mouse model, and that the treatment with an EP1 antagonist decreased ACF formation [33]. Schumacher et al. demonstrated that PGE2 exposure to human CRC cell lines promoted the dephosphorylation of cAMP response element-binding protein-regulated transcription co-activator 1 (CRTC1) to enhance CRTC1 transcriptional activity through EP1 and EP2 receptors signaling, resulting in the promotion of sporadic or colitis-associated colon cancer [34]. Sonoshita et al. reported that the important role of EP2 receptor by showing that homozygous deletion of the gene encoding EP2 reduced intestinal adenoma size and number in *Apc^Δ716^* mice [35].

In addition, EP3 has been shown to stimulate angiogenesis and tumor growth arising from implanted sarcoma cells in mice [36]. Fujino et al. reported that EP3 receptors could contribute to tumor cell metastasis by increasing cellular migration through the up-regulation of vascular endothelial growth factor receptor-1 (VEGFR-1) signaling [37]. However, other studies reported that EP3 was down-regulated in colorectal neoplasia and that AOM-induced tumorigenesis was accelerated in EP3-null mice, suggesting a tumor-suppressive role for EP3 in the intestine [38]. Macias-Perez et al. also reported that selective activation of EP3 could suppress tumor cell function of CRC cells in vitro and in vivo by activating a G12-RhoA pathway [39]. Therefore, the role of EP3 in colonic tumorigenesis might be controversial and further investigation is required. 

Chell et al. reported that EP4 was up-regulated during human CRC tumorigenesis in vivo [40]. Mutoh et al. reported that genetic or pharmacological inactivation of EP4 inhibited tumor growth in a mouse model of intestinal neoplasia [41]. Hsu et al. revealed that EP2 and EP4 were the major PGE2 receptors expressed on LoVo colon cancer cells and promoted cellular migration via the phosphoinositide 3-kinase (PI3K)/Akt pathway [42]. Wang et al. reported that PGE2 induced the expansion of cancer stem cells by activating nuclear factor kappa-light-chain-enhancer of activated B cells (NF-κB), via EP4-PI3K and EP4-mitogen-activated protein kinase (MAPK) signaling, which resulted in promotion of liver metastases in mice [43].

Taken together, most studies investigating the downstream signaling of PGE2 have shown that PGE2/EP signaling promotes the growth of CRC cells, although some reports showed data suggesting a tumor suppressive role of EP3 signaling in CRC. Further investigation is necessary for the complete understanding of PGE2/EP signaling in tumor cells. 

## 3. PGE2/EP Signaling in TME

**Figure 2:**
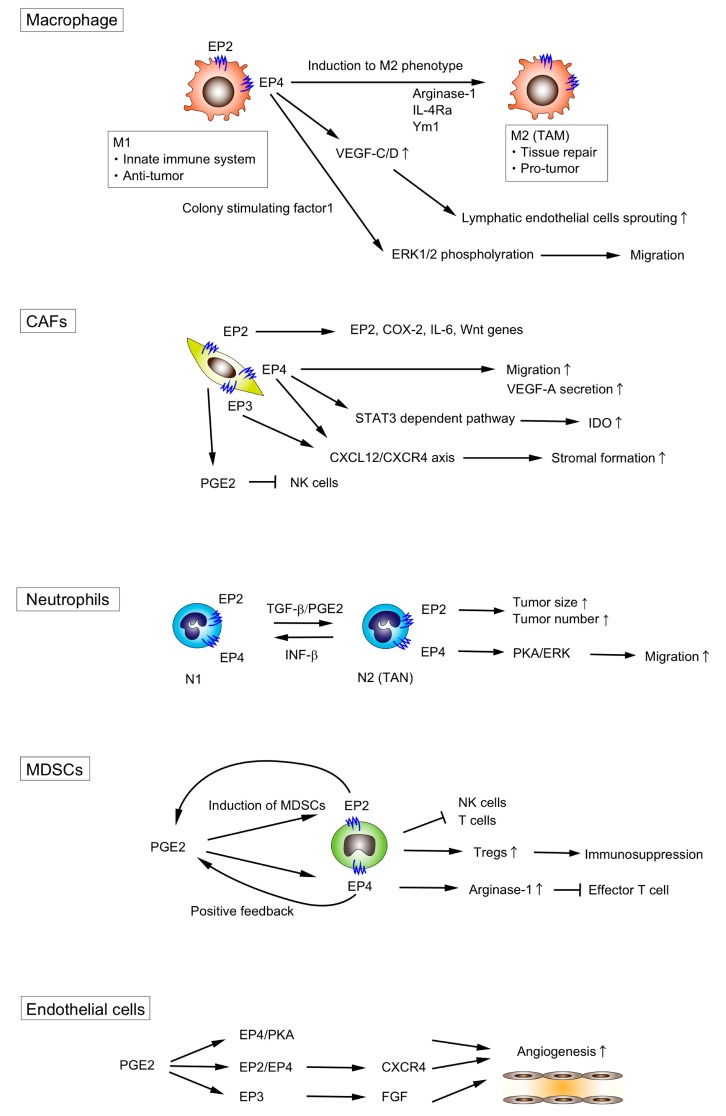
PGE2/EP signaling in TME. PGE2/EP signaling in each TME component promotes tumorigenesis by (i) switching the phenotype of macrophages and neutrophils from anti-tumor to pro-tumor, (ii) accelerating the migration of macrophages, CAFs and neutrophils, (iii) promoting lymphatic endothelial sprouting and angiogenesis, and (iv) suppressing the functions of T cells or NK cells. Arrows indicate positive regulation, while T-bars indicate negative regulation.

A number of studies have revealed the role of TME components such as macrophages, fibroblast, myeloid-derived suppressor cells (MDSCs), and neutrophils in the CRC tumorigenesis which promote the tumor growth by infiltrating into the tumor tissue and adjacent tissue [10,11,12]. Here, we highlight the role of PGE2/EP signaling of each component of the TME (Figure 2, Table 1).

### 3.1. Macrophages

Macrophages play pivotal roles in the host innate immune response against any pathogenic infections [76], and are one of the most dominant leukocyte residents found in the TME [77,78]. There are two types of macrophages: M1 macrophages and M2 macrophages. M1 macrophages act in the innate immune response against pathogenic infection, while M2 macrophages act in tissue repair and tumor progression [79]. In the context of TME, macrophages found in the TME are often referred to as tumor-associated macrophages (TAMs) which primarily belong to M2 phenotype. TAMs are one of the most abundant component of TME which represent up to 50% of the tumor cell mass [80,81,82]. TAMs promote tumor progression in the TME by directly accelerating tumor cell growth and angiogenesis, or by indirectly inducing the dysfunction of anti-tumor immune response [76,79,83,84]. The increased ratio of pro-tumor macrophages against anti-tumor macrophages is associated with decreased overall survival in Stage III CRC patients [85]. 

Inada et al., demonstrated that macrophages generate PGE2 via the upregulation of COX-2 by the mucins secreted from a colon cancer cell line LS180. They immunohistochemically analyzed the human colorectal cancer tissues and demonstrated that the localization of COX-2 expressing macrophages were located around the region in which mucins were detectable [86].

PGE2 signaling is known to play an important role in the polarization of the macrophages. PGE2 switches the phenotype of macrophages from anti-tumor M1 macrophages to pro-tumor M2 macrophages [44,45]. Eruslanov et al., reported that the overexpression of 15-PGDH in a mouse colon cancer cell line, CT26, switched the phenotype of intratumoral CD11b cells from M2-oriented TAMs to M1-oriented macrophages, suggesting that PGE2 can induce the differentiation of monocytes toward M2-type TAMs [46].

Wu et al. demonstrated that PGE2 could be a potent inducer of VEGF in M2 TAMs under hypoxic conditions [47]. Ratcliffe et al. examined the EP receptors expressed on macrophages and found that EP2 and EP4 were mainly expressed on macrophages [87]. Other groups also revealed that EP2 and EP4 receptors were expressed in cultured murine macrophage-like cell lines such as J774.1 and RAW264.7 [88,89].

Despite the fact that EP2 is expressed on macrophages, few studies have reported that EP2 signaling plays an important role in the functions of TAMs. Wu et al. assessed whether macrophage polarity was altered with loss of EP2, and revealed that the ratio of M1/M2 macrophage subtypes was not significantly changed with loss of EP2 expression [90]. 

On the other hand, the EP4 signaling has been reported to play a pivotal role in TAMs. Lala et al. showed that EP4 activation on macrophages upregulated VEGF-C/D production to stimulate lymphatic endothelial cells sprouting [48]. Digiacomo et al. reported that PGE2-EP4 signaling and colony-stimulating factor-1 signaling synergistically promoted the migration of macrophages via ERK1/2 phosphorylation [49]. PGE2/EP4 signaling could regulate the plasticity of the macrophages. Yasui et al. showed that treatment with EP4 agonist enhanced M2 polarization in wild-type peritoneal macrophages, whereas EP4-deficient macrophages were less susceptible to M2 polarization [50]. Chang et al. showed that the deletion of myeloid EP4 receptors led to the decreased expression of the M2 macrophage markers, arginase-1 and IL-4Rα in *APC^Min/+^* intestinal adenoma macrophages [51]. Zhang et al. also demonstrated that the expression of the M2 phenotype marker, Ym1, was decreased in the myeloid-specific EP4 knockout mice [52]. Albu et al. reported that the EP4 antagonist, E7046, reduced M2-like macrophages [53]. Barminko et al. also reported that EP4 signaling could induce the transition from M1 to M2 phenotype [54]. Collectively, these data suggest that EP4 rather than EP2 plays an important role in regulating the phenotype of macrophages in the downstream of PGE2.

On the contrary, another study has reported that PGE2/EP4 signaling activated inflammasome and induced M1 polarization of macrophages during the gram-negative bacteria infection [55]. It is reported that the role of PGE2 signaling is context dependent [90], and this is the result obtained from the setting of infection, which might affect the opposite results. 

### 3.2. Fibroblasts

Fibroblasts found in the TME are called as cancer-associated fibroblasts (CAFs) or tumor-associated fibroblasts (TAFs). CAFs are reported to account for a high proportion of tumor stroma (30–60%) [91]. They locate in the intratumoral stroma and the tumor-surrounding stroma.

There is considerable interest in understanding the biology of CAFs as they are recognized as a central element in the TME, with known roles in inflammation, tumor survival, metabolic reprogramming, and angiogenesis [92,93,94]. CAFs have been reported to accelerate tumor progression by secreting the extracellular matrix, remodeling their pro-inflammatory gene signature, and guiding cancer cells during invasion [95]. In stage II colorectal cancer, CAF-specific endoglin expression at invasive borders was associated with poor metastasis-free survival [96]. 

Although less is known about the role of PGE2/EP signaling in the activation or induction of CAFs, it is known that CAFs generate PGE2 [97]. Li et al. reported that CAFs suppressed natural killer (NK) cell functions through the production of PGE2 in vitro [56]. n stromal fibroblasts derived from mouse gastric cancer, PGE2 induced VEGF-A secretion, although the involvement of the downstream EP receptors was not analyzed [57]. Less is known about the role of EP receptor in CAFs, although Odaka et al. identified that EP2 and EP4 were expressed in lung fibroblasts [98]. 

Katoh et al. assessed the stromal formation of xenografts of Lewis lung carcinoma cells using knockout mice for each EP receptor, and found that the stromal formation was significantly suppressed in either EP3 or EP4 knockout mice, whereas neither EP1 nor EP2 knockout did affect stromal formation [59]. They demonstrated that PGE2 regulated stromal formation through EP3/EP4 receptor signaling to promote fibroblast recruitment via CXCL12/CXCR4 chemokine axis, and that EP3- or EP4-specific agonists stimulated CXCL12 expression from fibroblasts, while neither EP1 nor EP2 stimulation did, indicating that both EP3 and EP4 receptors on fibroblasts mediate CXCL12 induction elicited by endogenous PGE2.

Inada et al. reported that the VEGF-A secretion from the CAFs within melanoma xenografts was enhanced by PGE2 and this enhancement was significantly suppressed by an EP4 antagonist, which suggest that PGE2-EP4 signaling plays an important role in the secretion of VEGF-A from CAFs [60]. 

EP4 signaling and STAT3-dependent pathway in fibroblasts were reported to be involved in the upregulation of indoleamine 2,3-dioxygenase (IDO) expression in response to PGE2 released from human breast cancer cells [61]. In addition, Kock et al. showed that the inhibition of PGE2 production or EP4 antagonist treatment suppressed the migration of IL-1-stimulated dermal fibroblasts towards human neuroblastoma cells, which suggests PGE2 /EP4 signaling may promote the migration of fibroblast [62].

Although various mechanisms by which PGE2/EPs signaling regulates CAFs have been reported, it is necessary to keep in mind that all the findings shown here cannot be applied to the CAFs in CRC owing to the tissue-specific nature of EP expression. In the context of CRC, Ma et al. revealed that CAFs within CRC expressed EP2 to promote colon tumorigenesis by regulating the expression of inflammation- and growth-related genes in a self-amplification manner [58]. They showed that EP2 stimulation in cultured fibroblasts induced expression of EP2 itself, COX-2, IL6, and Wnt genes. As CAFs comprise the predominant stromal cell type, further work is necessary to elucidate the role of PGE2/EP signaling in the CAFs within CRC.

### 3.3. Neutrophils

Recent accumulating evidence has shown that some populations of neutrophils, known as tumor-associated neutrophils (TANs), could function as supporting tumor growth, invasion, and angiogenesis, although they have been classically considered to exhibit a defensive response against tumor cells [13]. Neutrophils have been originally viewed as the first-responders of the innate immune system against extracellular pathogens. However, recent evidence has added a new aspect on the function of neutrophils. Neutrophils are involved in the regulation of innate and adaptive immune systems, and can be polarized towards distinct phenotypes in response to environmental signals [99]. As with TAMs, recent studies have suggested that TANs also exhibit considerable plasticity and are capable of polarization into either an anti-tumorigenic “N1” phenotype or a pro-tumorigenic “N2” phenotype [99,100,101].

Although the effect of intratumoral neutrophils on the survival of CRC patients is still unclear, the increase of neutrophil count in peripheral blood (i.e., neutrophil-to-lymphocyte ratio (NLR)) has been shown to be related to poor clinical outcomes in CRC patients in several cohort studies including Stage I-III cancer, resectable or unresectable liver metastasis of CRC [13]. 

The phenotype of TANs depends on the signals encountered in the TME; TGF-β and interferon-β could regulate the plasticity of TANs [65,100]. Shaul et al. showed that PGE2 signaling functioned as a regulator between “N1” and “N2” phenotypes [63]. However, few studies have demonstrated which EP receptor can transduce the PGE2 signaling in TANs. 

Ma et al. demonstrated that EP2 deficiency in mice significantly decreased the size and number of intestinal tumors induced by AOM/DSS and that the number of infiltrating neutrophils in the colon was also decreased, although no significant changes in the number or size of intestinal tumors was observed in EP1 or EP3 knockout mice. They showed that PGE2/EP2 expression in infiltrating neutrophils was associated with ulcerative colitis, which suggest the role of EP2 signaling in the neutrophil-mediated inflammatory responses in the colon [58]. They also demonstrated the self-amplification loop consisting of COX-2/PGE2/EP2/NF-κB/COX2 in neutrophils. 

It was reported that TANs with the N2 phenotype are less migratory than those with the N1 phenotype. We previously demonstrated that PGE2/EP4 signaling plays a pivotal role in the migration of neutrophils [64]. PGE2/EP4 signaling in neutrophils activates PKA, which inhibits ERK to result in the suppression of the neutrophil migration. Therefore, PGE2/EP4 signaling might be involved in the induction of N2 neutrophils.

Taken together, little is known about the downstream signaling of PGE2 in the regulation of CRC-related TANs and further investigation is needed.

### 3.4. Myeloid-Derived Suppressor Cells (MDSCs)

MDSCs are defined as a heterogenous population of immature myeloid cells that have potent immunosuppressive properties against T cells and NK cells. Although they are classically defined as myeloid cells expressing the markers CD11b and Gr-1, the cellular nature of MDSCs is now better defined and includes two major subsets based on their phenotypic and morphological features: polymorphonuclear (PMN)-MDSC and monocytic (M)-MDSC. PMN-MDSC are defined as CD11b^+^CD14^−^CD15^+^ or CD11b^+^CD14^−^CD66b^+^ and M-MDSC as CD11b^+^CD14^+^HLA-DR^−/low^CD15^−^ [102].

The levels of MDSCs in the blood are positively correlated with clinical cancer stage and metastatic tumor burden in mice and patients [77,103]. Yang et al. demonstrated that MDSCs comprise approximately 5% of tumor volume in mice bearing MC-26 CRC cells, suggesting that MDSCs play an important role in the TME of CRC [104]. Veltman et al. showed that the treatment of tumor-bearing mice with a COX-2 selective inhibitor prevented the local and systemic expansion of MDSCs in vivo [105]. It is widely accepted that MDSCs contribute to cancer immune evasion by suppressing the functions of T and NK cells [106]. In mice injected with MC-26 CRC cell lines, Huang et al. demonstrated that MDSCs could mediate the development of regulatory T cells (Tregs), thereby suppressing anti-tumor immune function [107].

PGE2 has been reported to promote tumor progression by inducing the differentiation of MDSCs and enhancing MDSC-mediated immune suppression [65]. Sinha et al. found that all four EPs were expressed on MDSCs in BALB/c mice, and that EP2 was critical for MDSCs induction and immunosuppressive function [66]. Other work indicated that both EP2 and EP4 signaling might be involved in MDSCs induction [67]. In vitro studies showed that PGE2 induced arginase I expression via EP4 in MDSCs, which was involved in MDSC-mediated immune suppression by blocking effector T cell function [68]. Obermajer et al. demonstrated the EP2- and EP4-mediated positive feedback loops between COX-2 and PGE2 enhanced multiple aspects of MDSC function [108]. In comparison, there is little evidence to indicate the importance of EP1 and EP3 expression on MDSCs.

### 3.5. Other Types of Cells in the TME

Endothelial cells are also one component of the TME. Although PGE2 is known to regulate angiogenesis, the direct effect of PGE2 on endothelial cells is cell type- and context-dependent. Zhang et al. revealed that PGE2 promoted angiogenesis through the EP4/PKA signaling pathway in human lung microvascular endothelial cells (HMVECs) [69]. Another group reported that the PGE2 facilitate angiogenesis by increasing CXCR4 expression via EP2 and EP4 [70]. PGE2/EP3 signaling activated the non-receptor tyrosine kinase c-Src to activate matrix metalloproteases, which lead to the transactivation of fibroblast growth factor (FGF) receptors resulting in the angiogenic response in postcapillary venular endothelial cells [71]. 

Wang et al. reported that the expression level of programmed death-1 (PD-1) in infiltrating CD4^+^ and CD8^+^ T cells within lung cancer tissues was closely related to the PGE2/EP2 and PGE2/EP4 signaling pathways. They suggested that the activation of PGE2/EP2 and PGE2/EP4 signaling may positively regulate the expression level of PD-1 in infiltrating CD8^+^ T cells to result in the immune tolerance in the TME of lung cancer [72]. Holt et al. reported that PGE2 suppressed the cytotoxicity and cytokine production of NK cells via EP4 signaling in breast cancer-bearing mice [73].

PGE2 was also reported to promote tumor growth by inhibiting the accumulation and activation of conventional dendritic cells (DC) [74]. Indeed, PGE2 can alter the differentiation, maturation, and capacity of cytokine secretion of DCs, which results in the immune tolerance [75]. 

As discussed here, evidence about the roles of PGE2/EP signaling in the TME of various types of cancers has been accumulated. However, the evidence in the CRC settings remains insufficient. As the role of each TME component is context-dependent, it is necessary to accumulate more information about the PGE2/EP signaling in the CRC-specific TME. 

## 4. The Possibility of PGE2/EP Signaling in the TME as a Potential Therapeutic Target

As the TME plays an important role in the development and progression of cancer cells, a big effort has been made to develop new therapeutic strategies targeting the TME. Various trials targeting the TME components including extracellular matrix (ECM), hypoxia and acidosis, neovascularization, immune system, CAFs, and exosomes have been attempted [109]. 

Previous studies demonstrated that therapies targeting PGE2 by NSAIDs or COX-2 selective inhibitors were able to prevent CRC tumorigenesis, although severe cardiovascular side effects were observed in some patients [27,28]. Therefore, targeting the downstream PGE2/EP signaling could be a potential therapeutic strategy. In fact, phase 1 clinical study of E7046, an EP4 inhibitor that targets immunosuppressive myeloid cells in the TME, has already been started (NCT02540291: https://clinicaltrials.gov/ct2/show/study/NCT02540291). 

The limitation of the strategy targeting PGE2/EP signaling in the TME is that the EP agonists or antagonists would be administered systemically and these drugs affect not only the components in the TME but also other types of cells, including both cancer cells andt7878t normal cells. Therefore, it is necessary to assess the effect of drugs on other types of cells and to investigate the drug delivery system specifically to the TME components to avoid the undesired side effects. 

## 5. Conclusions

Accumulating evidence has shown that TME components, including macrophages, MDSCs, neutrophils, and fibroblasts, play an important role in the development and progression of CRC. PGE2 is important in the development and progression of CRC and affects not only cancer cells but also cells in the TME. The current therapies targeting PGE2 using NSAIDs or COX-2 inhibitors have failed due to the global prostanoid suppression resulting in the severe adverse effects despite their effects to prevent tumorigenesis. Therefore, therapies targeting the specific downstream molecules of PGE2 signaling could be a promising approach. This review highlighted the role of each EP receptor in the TME of CRC tumorigenesis and their therapeutic potential against CRC. 

## Figures and Tables

**Table 1:** The role of PGE2/EP signaling in TME component.

TME Component	Signaling	Effect	Reference
Macrophages	PGE2	Enhance M2 polarization	[44,45,46]
		Induce VEGF production from M2 macrophages	[47]
	EP4	Stimulate lymphatic endothelial sprouting through the Increase of VEGF-C/D	[48]
		Promote the migration of macrophages via ERK1/2	[49]
		Enhance M2 polarization	[50,51,52,53,54]
		M1 polarization during gram-negative bacteria infection	[55]
Fibroblasts	PGE2	Suppress NF cell function	[56]
		Promote VEGF-A production	[57]
	EP2	Induce EP2, COX-2, IL-6 and Wnt genes expression	[58]
	EP3/EP4	Promote stromal formation via CXCL12/CXCR4	[59]
	EP4	Promote VEGF-A production	[60]
		Upregulate IDO expression	[61]
		Promote migration	[62]
Neutrophils	PGE2	Enhance N2 polarization	[63]
	EP4	Promote migration via PKA/ERK signaling	[64]
MDSCs	PGE2	Induce the differentiation of MDSCs	[65]
	EP2	Induce MDSCs	[66]
	EP4	Induce MDSCs	[67]
		Induce Arginase I expression which is critical for immune suppression of T cells	[68]
Endothelial cells	PGE2	Promote angiogenesis through the EP4/PKA signaling	[69]
	EP2/EP4	Promote angiogenesis through CXCR4	[70]
	EP3	Promote angiogenesis through the upregulation of Src	[71]
Lymphocytes	EP2/EP4	Incduce immune tolerance by increasing PD-1 expression in infiltrating CD8+ cells	[72]
NK cells	EP4	Suppress NK cell functions	[73]
DC cells	PGE2	Inhibit the accumulation and the activation of DC cells	[74,75]

## Abbreviations

ACF	Aberrant crypt foci
cAMP	Cyclic adenosine monophosphate
AOM	Azoxymethane
CAFs	Cancer-associated fibroblasts
COX-2	Cyclooxygenase-2
CRC	Colorectal cancer
CRTC1	Camp response element-binding protein-regulated transcription co-activator 1
DSS	Dextran sulfate sodium
ERK	Extracellular signal-regulated kinase
GPCR	G protein-coupled receptor
IBD	Inflammatory bowel disease
IDO	Indoleamine 2,3-dioxygenase
MDSCs	Myeloid-derived suppressor cells
NF-κB	Nuclear factor kappa-light-chain-enhancer of activated B cells
NK cells	Natural killer cells
NSAIDs	Non-steroidal anti-inflammatory drugs
PI3K	Phosphoinositide 3-kinase
PKA	Protein kinase A
RNI	Reactive nitrogen intermediates
ROS	Reactive oxygen species
TAFs	Tumor associated fibroblasts
TAM	Tumor-associated macrophages
TAN	Tumor associated neutrophils
TME	Tumor microenvironment
VEGF	Vascular endothelial growth factor
VEGFR	Vascular endothelial growth factor receptor
